# Stress in utero: prenatal dexamethasone exposure causes greater structural gliovascular alterations in female offspring than in males

**DOI:** 10.3389/fnins.2025.1539867

**Published:** 2025-03-24

**Authors:** Magda Ferreira-Rodrigues, Inês Santos Sousa, Filipa I. Baptista, Vanessa Coelho-Santos

**Affiliations:** ^1^PhD Programme in Experimental Biology and Biomedicine, Institute for Interdisciplinary Research, University of Coimbra, Coimbra, Portugal; ^2^Center for Neuroscience and Cell Biology (CNC-UC), Institute for Interdisciplinary Research, University of Coimbra, Coimbra, Portugal; ^3^Institute for Nuclear Sciences Applied to Health (ICNAS), University of Coimbra, Coimbra, Portugal; ^4^Coimbra Institute for Biomedical Imaging and Translational Research (CIBIT), University of Coimbra, Coimbra, Portugal; ^5^Faculty of Medicine, Institute of Physiology, University of Coimbra, Coimbra, Portugal; ^6^Faculty of Medicine, Coimbra Institute for Clinical and Biomedical Research (iCBR), University of Coimbra, Coimbra, Portugal; ^7^Center for Innovative Biomedicine and Biotechnology (CIBB), University of Coimbra, Coimbra, Portugal; ^8^Clinical Academic Center of Coimbra (CACC), Coimbra, Portugal; ^9^Institute of Pharmacology and Experimental Therapeutics, University of Coimbra, Coimbra, Portugal

**Keywords:** prenatal stress, glucocorticoids, developing brain, gliovascular, cerebrovascular

## Abstract

From early in life, experiences like prenatal stress profoundly affect long-term health and behavior. Fetal exposure to increased levels of glucocorticoids (GC), via maternal stress or through antenatal corticosteroid therapy (commonly used in women at risk of preterm birth), can disrupt brain development and raise the susceptibility to psychiatric disorders. Previous studies on prenatal exposure to synthetic GCs, such as dexamethasone (DEX), revealed impairments in neurogenesis and dendritic spine development. However, the impact of prenatal stress, specifically antenatal DEX exposure, on the gliovascular interface remains unclear. This interface, involving the relationship between astrocytes and blood vessels, is essential for healthy brain development. Astrocytic endfeet coverage and organization are crucial features of the gliovascular interface, and in this study, we evaluated these aspects through aquaporin-4 (AQ4) expression and organization along the lectin labelled-vasculature. At Postnatal Day 14, no differences in AQ4 expression were observed between males and females. However, prenatal stress induced by DEX exposure (50 μg/kg was administered subcutaneously to pregnant mice through gestational days 16, 17 and 18) significantly impacted this structure in females but not in males. Specifically, in female offspring prenatally exposed to DEX, AQ4 expression was significantly upregulated in the hippocampus, and its rearrangement was observed in the prefrontal cortex. A comparison of vascular density between male and female brains showed no significant sex differences in any analyzed regions, though male cerebellar vessel segments were shorter. Interestingly, prenatal stress caused morphological alterations in female brains, including increased vessel tortuosity, while no such changes were seen in males. In the hippocampus, prenatal DEX exposure reduced vessel segment length in males but did not affect females. In the cerebellum, DEX exposure increased vessel segment length in females. This study highlights sex-specific differences in the impact of prenatal stress on the gliovascular structure across various brain regions, suggesting AQ4 as a potential molecular target relevant to depressive-like behaviors in female offspring. Future studies are needed to correlate the gliovascular structural alterations found with functional disturbances and sex-specific mental health issues.

## 1 Introduction

During pregnancy, glucocorticoids (GC) such as cortisol naturally increase in the mother’s body. These hormones, important basal and stress-related homeostasis regulators, cross the placenta to prepare the fetus for postnatal life (Krontira et al., 2020) helping with crucial aspects of fetal organ maturation, especially the development of the lungs. However, stressful events during pregnancy, such as financial hardships, exposure to natural disasters, or major life changes, elevate GC levels. This influences the programming of the hypothalamic–pituitary–adrenal (HPA) axis (Sheng et al., 2023), impacting in particular the fetal brain development (Sheng et al., 2023) and potentially increasing vulnerability to stress-related disorders later in life (reviewed in Bock et al., 2015).

In pregnancies with a risk of preterm delivery, the fetus may not have enough time to undergo these developmental steps, resulting in high risks of respiratory distress syndrome, intraventricular hemorrhage and other complications (Ballabh, 2010). To mitigate these risks, synthetic GC like dexamethasone (DEX) therapy, is widely used in clinical settings for women at risk of preterm labor. Administered in controlled doses, they accelerate lung and organ development, improving survival and reducing complications like brain hemorrhage in preterm infants. While synthetic GCs reduce neonatal mortality and morbidity (Zhao et al., 2022), repeated or high doses can harm fetal brain development (Shimada et al., 2022; Mino et al., 2023), increasing the risk of psychiatric disorders through structural and functional abnormalities (Rodrigues et al., 2012; Oliveira et al., 2006; Rim et al., 2022). Synthetic GC administration adds complexity by raising maternal blood pressure and glucose levels while also intensifying stress from preterm birth and treatment.

The increased levels of GCs, resulting from maternal stress or the administration of GC therapy during gestation, induce fetal stress that significantly affects offspring mental health (reviewed by Krontira et al., 2020). In adulthood, these mental health challenges manifest in sex-specific behaviors: schizophrenia-related behaviors in males and anxious-depressive like symptoms in females, alongside social deficits increased emotional reactivity and drug-seeking behaviors (Rodrigues et al., 2012; Oliveira et al., 2006; Rim et al., 2022; Oliveira et al., 2012; Caetano et al., 2017; Borges et al., 2013). Prenatal exposure to GCs, including DEX, has been linked to various brain-region-specific structural alterations. These include volumetric atrophy of the nucleus accumbens (Rodrigues et al., 2012), increases the bed nucleus of the stria terminalis volume, while reducing amygdala volume (Oliveira et al., 2012). At the cellular level, prenatal GCs impair neurogenesis (Tsiarli et al., 2017) and radial migration of post-mitotic neurons during the development of the cerebral cortex (Fukumoto et al., 2009), hinder dendritic spine development (Oliveira et al., 2012; Tanokashira et al., 2012), compromise neuronal survival (Crochemore et al., 2005) and lead to morphological alterations in axons and dendrites of developing neurons (Pinheiro et al., 2018). Fetal stress induced by GCs exposure also affects microglia, the brain’s immune cells, potentially explaining sex-specific vulnerabilities to psychiatric disorders (Rim et al., 2022; Caetano et al., 2017; Gaspar et al., 2021). Furthermore, GCs also influence gene expression in astrocytes, altering their function (Carter et al., 2013; Carter et al., 2012) and impacting astrocytic signaling pathways essential for neurovascular development and synaptic formation, including impairments in the fibroblast growth factor 2- FGFR1-fibroblast growth factor receptor 1 (FGF2-FGFR1) axis, which may contribute to long-term cognitive deficits (Choi et al., 2022).

Stress in adulthood has been shown to impact brain vasculature negatively (Collignon et al., 2024; Welcome and Mastorakis, 2020), compromising of integrity of the blood–brain barrier (BBB), which plays a crucial role in regulating the passage of molecules and cells between the bloodstream and the brain. Both vasculature and its barrier integrity are vital for brain development and function (Coelho-Santos and Shih, 2020). Disruptions in the BBB during critical developmental periods can have long-lasting consequences on neuronal connectivity, synaptic plasticity, and overall brain function (Ouellette et al., 2020). These alterations influence stress responses, affecting resilience and susceptibility to psychiatric conditions (Dion-Albert et al., 2022; Dudek et al., 2020; Najjar et al., 2017). Despite the scarcity of research on prenatal stress, emerging evidence hints at its potential influence on brain vasculature. Rats exposed to prenatal DEX (subcutaneous injection 100 mg/kg on G14-21) present reduced vascular area in the CA3 region of the hippocampus in adulthood compared to controls (Neigh et al., 2010). In a mouse model of prenatal DEX exposure, it was found that at postnatal day (P) 4 there is a significant downregulation of the tight junction protein claudin-5 in total brain samples after multiple intraperitoneal injections with DEX (0.1 mg/kg at G15-17). Immunofluorescence images of the tight junction claudin-5 and the endothelial marker revealed changes in vessel morphology as well (Neuhaus et al., 2015). A previous study in sheep showed that single maternal treatment with DEX (four 6 mg intramuscular injections every 12 h over 48 h) led to an increase of claudin-5 in the cerebral cortex and upregulation of occludin in the cerebellum. Multiple exposures (once a week for 5 consecutive weeks) did not impact claudin-5 expression levels in the cerebellum but upregulated occludin in the cerebral cortex and cerebellum (Sadowska et al., 2010), suggesting that changes in the brain are region-specific. Additionally, prenatal GC treatment inhibited the vascular endothelial growth factor and increased the transforming growth factor-beta levels, which resulted in angiogenic inhibition and enhancement of pericyte coverage (Vinukonda et al., 2010).

Interestingly, the effects of prenatal stress on the gliovascular interface remain elusive and require further clarification. This interface, where astrocytes intimately interact with blood vessels in the brain, is a critical hub for maintaining the BBB integrity (Gilbert et al., 2019) and a participant in the neurovascular coupling (Freitas-Andrade et al., 2023), which ensures proper blood flow in response to neuronal activity. Therefore, unraveling the complex interplay between astrocytes and vessels is crucial for understanding the minutiae of healthy brain development. To address this gap and investigate potential gliovascular alterations linked to prenatal stress, we subcutaneously administered physiological saline or 50 μg/kg of DEX to pregnant mice from gestational days (GD) 16 to 18 and analyzed offspring brain tissue at P14 using immunohistochemistry techniques in several regions. This study invites further investigation into how prenatal stress shapes infant gliovascular development, potentially influencing adult sex-specific mental health issues.

## 2 Methods

### 2.1 Animals and experimental design

**Figure 1:**
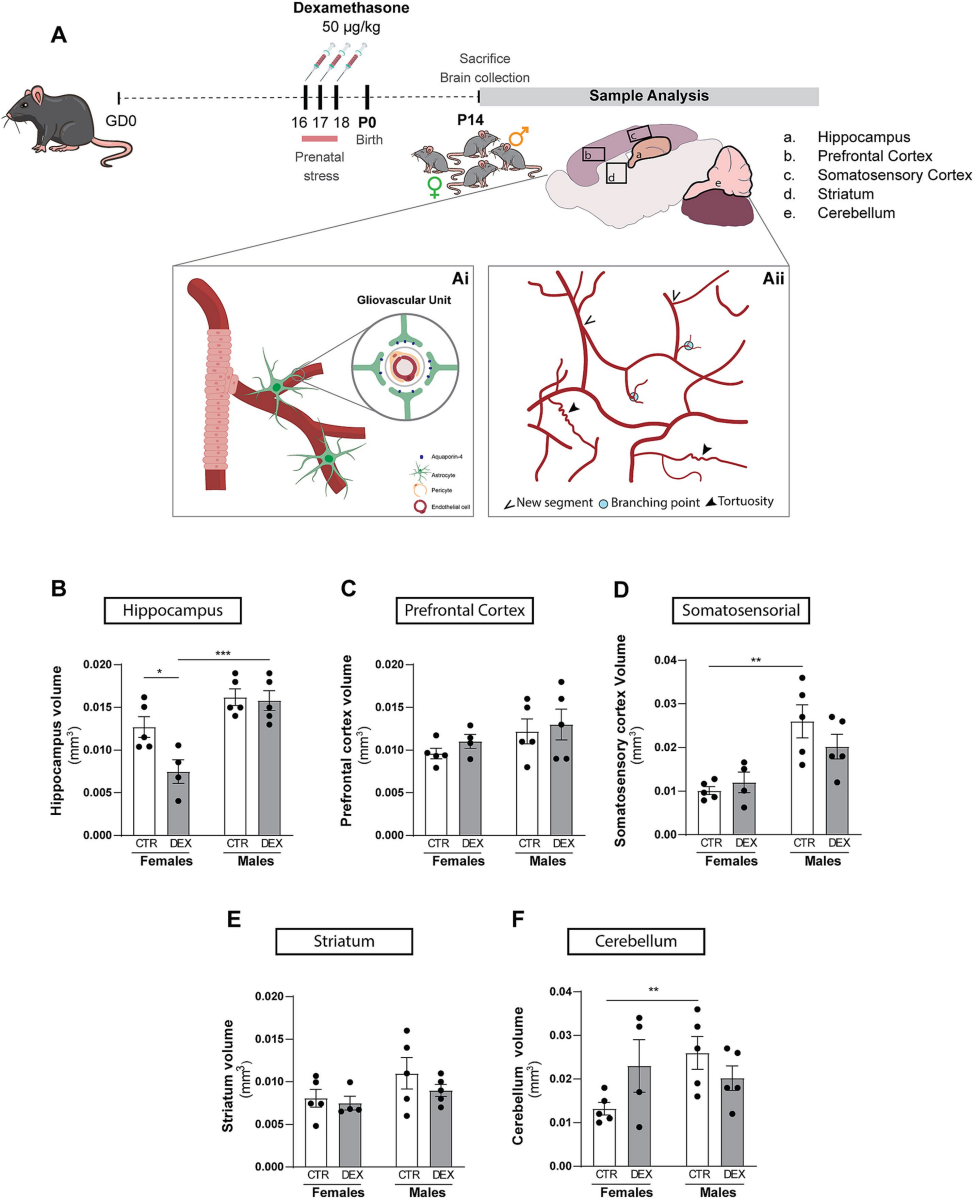
Schematic representation of study methodology. **(A)** This schematic provides a visual overview of the research plan executed, outlining the key methods employed and the overarching objectives of the study (Ai and Aii). The analysis was focused on the hippocampus (a), prefrontal cortex (b), somatosensory cortex (c), striatum (d), cerebellum (e). **(B–F)** Quantification of the impact of prenatal stress treatment on various brain regions volume in female and male offspring. Results are presented as the mean ± SEM, with *n* = 3–5 animals. **p < 0.05, a two-way ANOVA was conducted to test the effects of prenatal stress (CTR vs. DEX) and sex (female vs. male).

Female C57BL/6 J mice weighing 19–25 g at reproductive age were housed in certified facilities, with temperature and humidity-controlled environment, under a 12 h:12 h light–dark cycle and with *ad libitum* access to water and food. Female mice were mated with males (1 male per female) for a period of 24 h. Male bedding was added to female’s cages the day prior male presentation. Gestational day 0 (GD0) was considered the day after mating day. Dams were arbitrarily assigned to control (CTR) or dexamethasone (DEX) experimental groups. DEX at the dose of 50 μg/kg or saline (vehicle) was administered subcutaneously to pregnant mice on GD16, 17 and 18 and has been validated to induce behavioral alterations associated with prenatal stress, including depressive-like behavior in female offspring, as previously described (Rim et al., 2022). One pup of each sex was selected from each litter of CTR or DEX dams and sacrificed on P14. Figure 1A illustrates a graphical overview of the experimental arrangement.

### 2.2 Sample collection

At P14, female (F) and male (M) offspring mice were deeply anesthetized with an intraperitoneal injection of ketamine (50 mg/kg; Nimatek, Dechra, United Kingdom) and xylazine (2 mg/kg; Sedaxylan, Dechra, UK). Animals were then transcardially perfused with 0.1 M phosphate-buffered saline (PBS, in mM: 137 NaCl, 2.7 KCl, 10 Na_2_HPO_4_, 1.8 KH_2_PO_4_, pH 7.4), followed by 4% paraformaldehyde (PFA) in 1% PBS. Brains were postfixed in 4% PFA, transferred to a solution of 30% sucrose in PBS-1% and stored at 80°C until processing.

### 2.3 Free-floating immunohistochemistry

Sagittal sections (100 μm) were obtained using a cryostat (Leica CM3050S) and submerged in PBS containing 0.01% sodium azide (Thermo Fisher Scientific, 26,628–22-8), then stored at 4°C until further use. Prior to immunostaining, slices underwent a 1 h incubation at room temperature (RT) in a blocking solution consisting of 0.1 M PBS supplemented with 5% (v/v) goat serum (Sigma; G9023) and 0.3% (v/v) Triton X-100 (Thermo Fisher Scientific, BP151-100). Subsequently, slices were transferred to well plates containing primary antibodies (Lectin- Fluorescein, 1:250, Vector Labs, FL-1171-1; Aquaporin 4-CoraLite 647, 1:250, Proteintech, CL647-16473) diluted in 0.1 M PBS with 5% (v/v) goat serum and 0.2% (v/v) Triton X-100 and incubated at 4°C for 48 h. Following this, slices were triple rinsing for 15 min each with 0.1 M PBS, succeeded by three 5 min rinses with 0.1 M PBS, under gentle agitation. Post-immunostaining, slices were mounted, air-dried, and sealed with DAPI Fluoromount-G (Invitrogen, 00–4,959-52) before imaging. Brain slices for imaging were selected from the same sagittal plane to ensure consistent comparisons across samples within regions, and one to three brain slices were analyzed. Whole slide imaging was acquired using a Slide scanner (Carl Zeiss Axio Scan.Z1) equipped with a 20x objective (Plan-Apochromat 20x/0.8 M27), capturing the maximum z-stack images feasible (scaling per pixel 0.325 μm x 0.325 μm x 2.500 μm). For high resolution imagens slices were mounted in liquid antifade Abberior Mount Medium (Abberior, MM-2009-1ML) and 2D immunofluorescence images were acquired on an Abberior Stimulated Emission Depletion (STED) microscope equipped with a STEDYCON, equipped with 100x oil immersion objective (Nikon Plan Apo 100x/ 1.45 oil, w.d: 0.13 mm).

### 2.4 Analysis of histological data

***Volumetric Analysis of Brain Regions and Ventricles***: To quantify the volume of specific brain regions the following structures were selected: hippocampus, prefrontal cortex, somatosensory cortex, striatum, and cerebellum. Additionally, the lateral ventricles were included for volumetric analysis. To ensure comparability, the same brain sagittal plane was used for this analysis in each sample. ImageJ (Fiji) software was used to measure the entire area of each region within the brain sagittal plane, and the maximum number of focused z-stacks was selected to calculate the volume of each region. A total of 1–3 fields of view (FOV) were analyzed per animal to quantify changes in volume across the different brain regions. The volume of each region was calculated by multiplying the measured area by the number of z-stacks.


$$\mathrm{Region}\;\mathrm{volume}\;\left(mm^{3}\right)=\frac{\mathrm{Area}\;\mathrm{region}}{\mathrm{Total}\;n\;\mathrm{of}\;z\;\mathrm{stacks}}$$

***Aquaporin 4 analysis***: The total expression of AQ4 was quantified within the predefined region of interest (ROI). Mean gray value and area measurements of the AQ4 channel were obtained from these ROIs. Background subtraction was performed by averaging the mean gray values from two background regions. Then, background value was subtracted from the mean gray value of AQ4. Image volume was calculated by multiplying the ROI area by the number of z-stacks used in each image (mm^3^). Finally, to calculate the total AQ4 expression the mean gray value obtained was divided by the volume (Fluorescence AU/mm^3^). A total of 1–3 fields of view (FOV) were analyzed per animal to quantify changes in AQ4 expression across the different brain regions.


$$\begin{array}{l} Aq4\;\exp\mathrm{ression}\;\left(\mathrm{Fluorescence}\;\mathrm{AU}/mm^{3}\right)=\\ \frac{\mathrm{Mean}\;\mathrm{Gray}\;\mathrm{Value}-\bar{x}\;\left(\mathrm{Brackground}\;\mathrm{mean}\;\mathrm{gray}\;\mathrm{values}\right)}{\mathrm{Area}\times \mathrm{number}\;\mathrm{of}\;z\;\mathrm{stacks}} \end{array}$$

In our study, we also investigated the colocalization of aquaporin 4 (AQ4) with lectin to demonstrate the association between astrocyte endfeet and blood vessels. Utilizing the ImageJ (Fiji), fluorescence images obtained from the Slide Scanner were converted into 2D images through max intensity Z-projection. Subsequently, a ROI was delineated, covering most of the brain regions, from prefrontal, striatum, somatosensory cortex, hippocampus and cerebellum. Colocalization analysis of fluorescence microscopy images were conducted using BIOP’s (Bioimaging and Optics Platform) version of JACoP (Just Another Colocalization Plugin; Bolte and Cordelières, 2006). The assessment involved measuring Pearson’s Correlation Coefficient. Pearson’s correlation coefficient was highlighted as it quantifies the strength and direction of the linear relationship between two variables, specifically the image pixels of fluorescence, providing insights into the colocalization patterns of AQ4 and lectin in astrocyte endfeet and vessels.

***Vasculature analysis***: To assess the impact of DEX on the vasculature, small ROIs were isolated from the previously described areas, utilizing the lectin channel. Prior to analysis images were converted into grayscale. Vessel tracing was meticulously conducted using the hand-free tracing tool in ImageJ, facilitating the creation of overlays on the z-stack images of vessels. Subsequently, various parameters were quantified to evaluate vascular morphology. The length of vessels was measured, and the number of branches was determined by the newly added traces during vessel tracing. Branching points were also carefully annotated whenever observed during the tracing process. In addition to the quantitative assessments, a qualitative analysis of vessel tortuosity was performed. Images were categorized based on the degree of tortuosity observed, with three distinct levels identified: “None” indicating no visible alterations in vessel morphology; “Low” denoting the presence of less than five vessels exhibiting tortuosity; and “High” indicating five or more vessels displaying noticeable roughness or tortuosity. A total of 1–3 fields FOV were analyzed per animal to quantify changes in the vasculature across the different brain regions. Finally, the results were presented based on the volume of each image (Area x number of z stacks). Representative images were acquired using a Zeiss LSM 710 confocal microscope with a Plan-Apochromat 20x/0.8 objective. An equal number of z-stack images were acquired for each brain region.

***Exclusion criteria***: From the slide scanner, z-stacks that exhibited blurriness in the images were excluded from further analysis.

### 2.5 Statistics

Data analysis was conducted using GraphPad Prism 10 software. Graphical representations show mean values accompanied by the standard error of the mean (SEM). A two-way ANOVA was conducted to test the effects of drug treatment (CTR *vs* DEX) and sex (female *vs* male). Statistical significance was set at *p* < 0.05. Further details regarding statistical tests are provided in the figure legends and Supplementary Tables S1–S5.

## 3 Results

### 3.1 Brain region volume alterations induced by prenatal stress in the offspring

Given the significant sex differences previously published in both brain development and behavioral outcomes after DEX exposure (Rodrigues et al., 2012; Oliveira et al., 2006; Rim et al., 2022; Oliveira et al., 2012; Caetano et al., 2017; Borges et al., 2013), we sought to investigate the impact of sex during prenatal stress conditions on the volumes of the brain regions analyzed in this study (Figures 1B–F). Early in development, male mice typically exhibit larger brain regions (Qiu et al., 2018; Tkáč et al., 2023). Our results tend to align with that statement, demonstrated by a tendency for an increased volume in males’ hippocampus (Figure 1B) and striatum (Figure 1E), with a significant difference observed in somatosensory cortex (*p* = 0.0014; Figure 1D) and cerebellum (*p* = 0.0410; Figure 1F), the prefrontal cortex (Figure 1C) showed no tendency. Male and female hippocampal offspring responded differently to DEX maternal treatment (*p* = 0.0004; Figure 1B). Prenatal stress reduced hippocampal volume in females (*p* = 0.0165; Figure 1B) but not in males. Curiously, the volume remained unchanged after prenatal stress conditions in prefrontal cortex (Figure 1C), somatosensory cortex (Figure 1D), striatum (Figure 1E) and cerebellum (Figure 1F). These findings provide further evidence about the importance of sex in shaping brain development and highlight the different impact of prenatal stress on brain regions.

### 3.2 Prenatal stress exposure affects the development of AQ4 in the offspring brains

AQ4, a crucial protein residing in the endfeet of astrocytes, plays a vital role. These astrocytic extensions tightly wrap around brain microvessels, acting as gatekeepers for water transport and maintaining the delicate homeostasis of fluids within the brain (Salman et al., 2022).

**Figure 2:**
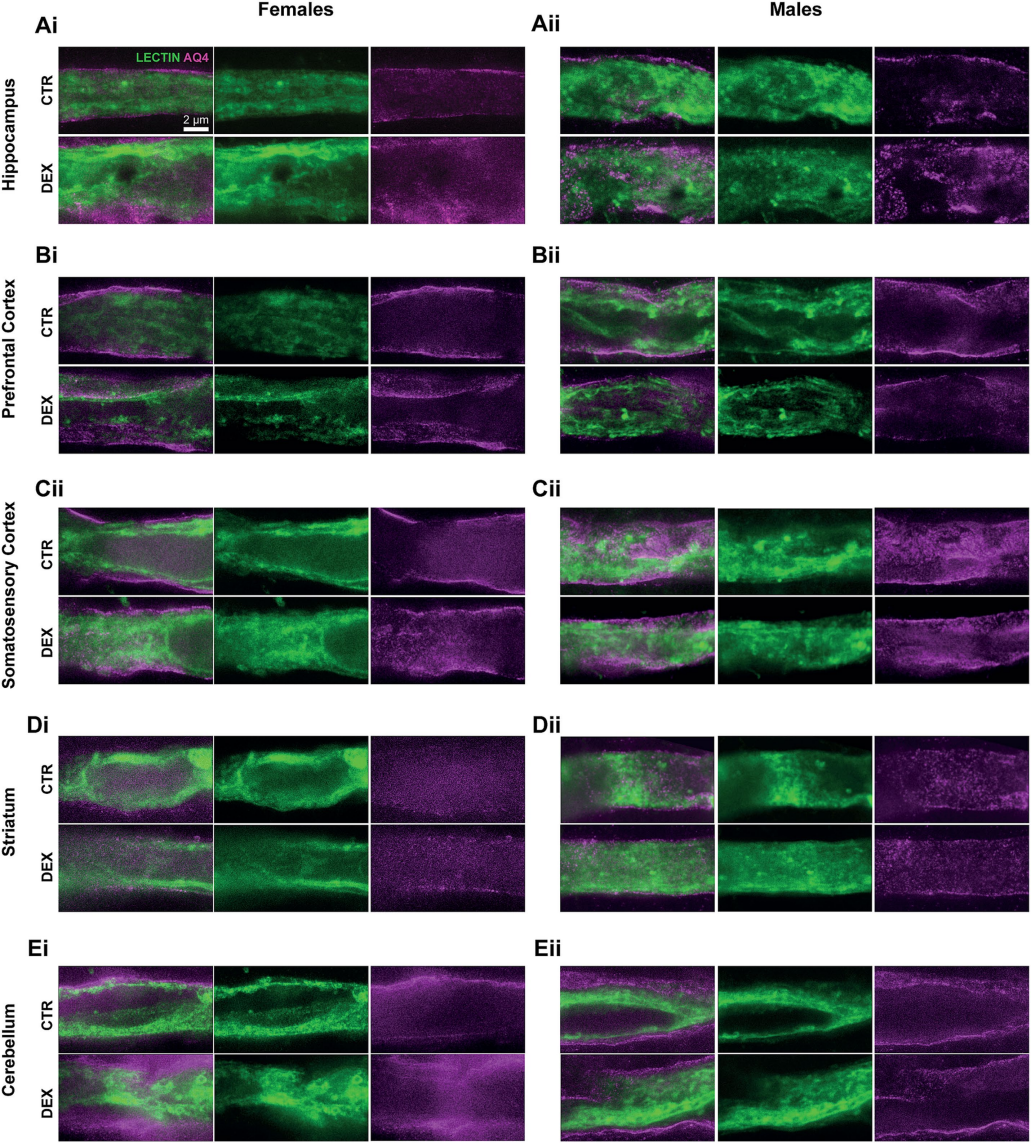
Representative high-resolution STED images of vessels and AQ4 coverage. Images show astrocytic endfeet coverage surrounding microvessels (lectin, green) and AQ4 protein (magenta) localization within these endfeet. Data are shown for females (i) and males (ii) in the hippocampus **(A)**, prefrontal cortex **(B)**, somatosensory cortex **(C)**, striatum **(D)**, and cerebellum **(E)**.

**Figure 3:**
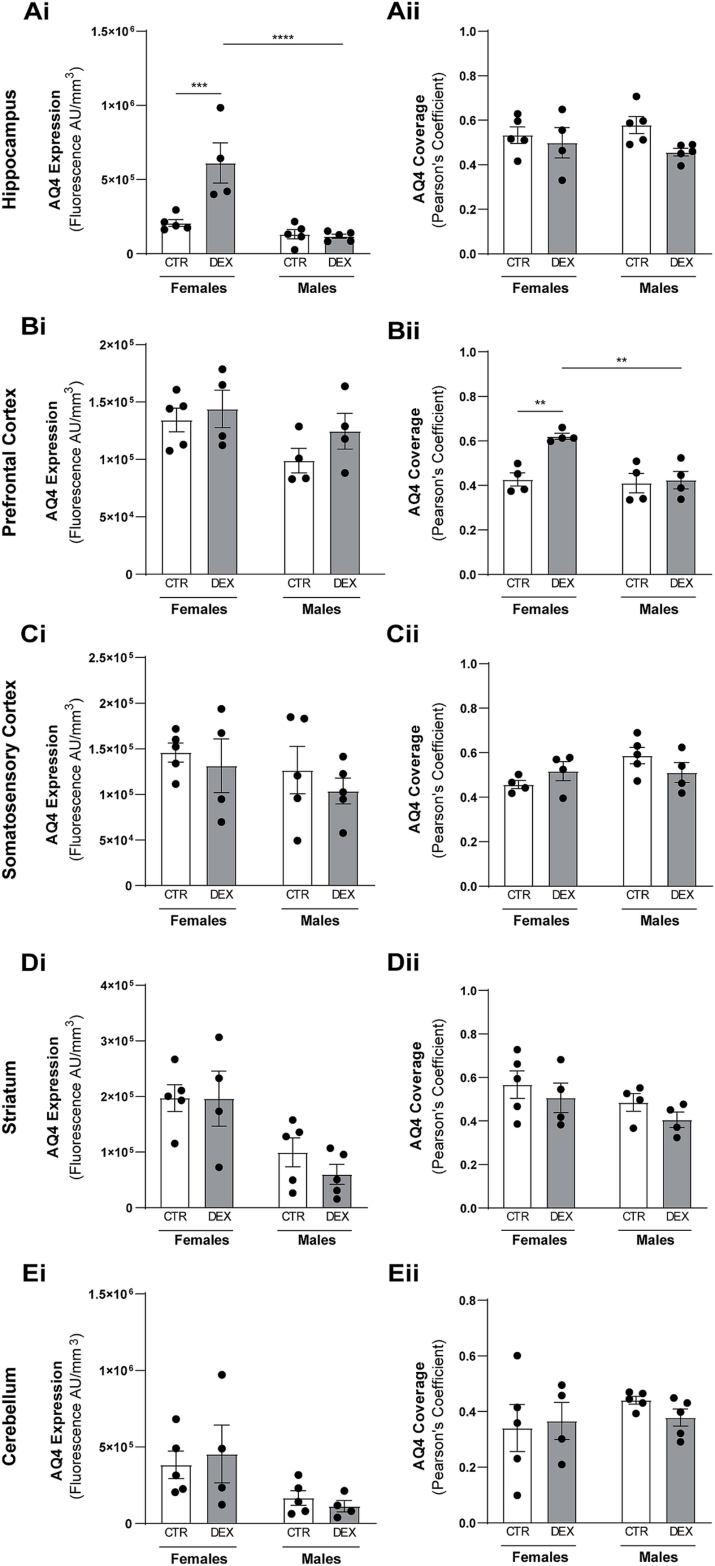
Dexamethasone prenatal exposure alters aquaporin-4 expression and colocalization with microvessels in females. Quantitative analysis of total aquaporin-4 (AQ4) expression levels and AQ4 colocalization with microvessels (Pearson’s coefficient) in the hippocampus **(A)**, prefrontal cortex **(B)**, somatosensory cortex **(C)**, striatum **(D)**, and cerebellum **(E)**. Results are presented as the mean ± SEM, with *n* = 4–5 animals. ***p < 0.0002, ****p < 0–0001 a two-way ANOVA was conducted to test the effects of prenatal stress (CTR vs. DEX) and sex (female vs. male).

In the hippocampus, although there was a tendency for different volumes between sexes, this did not reflect in the gliovascular unit, where females and males presented the same AQ4 expression levels and vascular coverage in this brain region (Figures 2A, 3Ai) in physiological conditions. However, a DEX sex-specific response was evident, with females and males exhibiting significantly distinct responses to prenatal stress (*p* < 0.0001; Figure 3Ai). In hippocampus of the females, we observed a significant increase in AQ4 protein expression levels (*p* = 0.0007; Figure 3Ai) without affecting the vascular coverage (Figure 3Aii). In contrast, prenatal stress exposure did not alter hippocampal AQ4 expression in males, as no significant changes were observed in AQ4 levels or its colocalization with the vasculature (Figures 3Ai,ii). In the prefrontal cortex of the female offspring, we noticed a significant increase in the coverage of blood vessels by AQ4 (*p* = 0.0027; Figures 2Bi, 3Bii); yet, the total amount of AQ4 protein remained unchanged (Figure 3Bi). This suggests a change in the distribution of existing AQ4 in the prefrontal cortex of females prenatally exposed to stress. Regarding male offspring, prenatal stress treatment did not impact prefrontal cortex AQ4 expression (Figures 2Bii, 3Bi) or distribution within the vessels (Figure 3Bii). A substantial sex difference in response to the treatment was observed in respect to AQ4 colocalization with vessels (*p* = 0.0030; Figures 2B, 3Bii), with females being more affected by prenatal stress. Another region demonstrating sex-specific effects of prenatal stress exposure was the striatum (Figure 2D; Supplementary Table S5). There are no physiological sex differences among AQ4 expression (Figure 3Di) or colocalization (Figure 3Dii). Interestingly, in females and males prenatally exposed to stress, the AQ4 protein levels (Figures 3Ci,Ei), and its vascular coverage (Figures 2C,E, 3Cii,Eii) prevailed unaltered at the somatosensory cortex and cerebellum comparing with controls.

In summary, our findings indicate that prenatal stress disrupts the normal developmental distribution and expression levels of AQ4 in the hippocampus, but only in females.

### 3.3 Effects of maternal stress on the development of the cerebrovascular network

To clarify if prenatal stress exposure could impact the vascular structure, we analyzed the 3D morphology of the cerebrovascular network in detail (Figure 1Aii).

**Figure 4:**
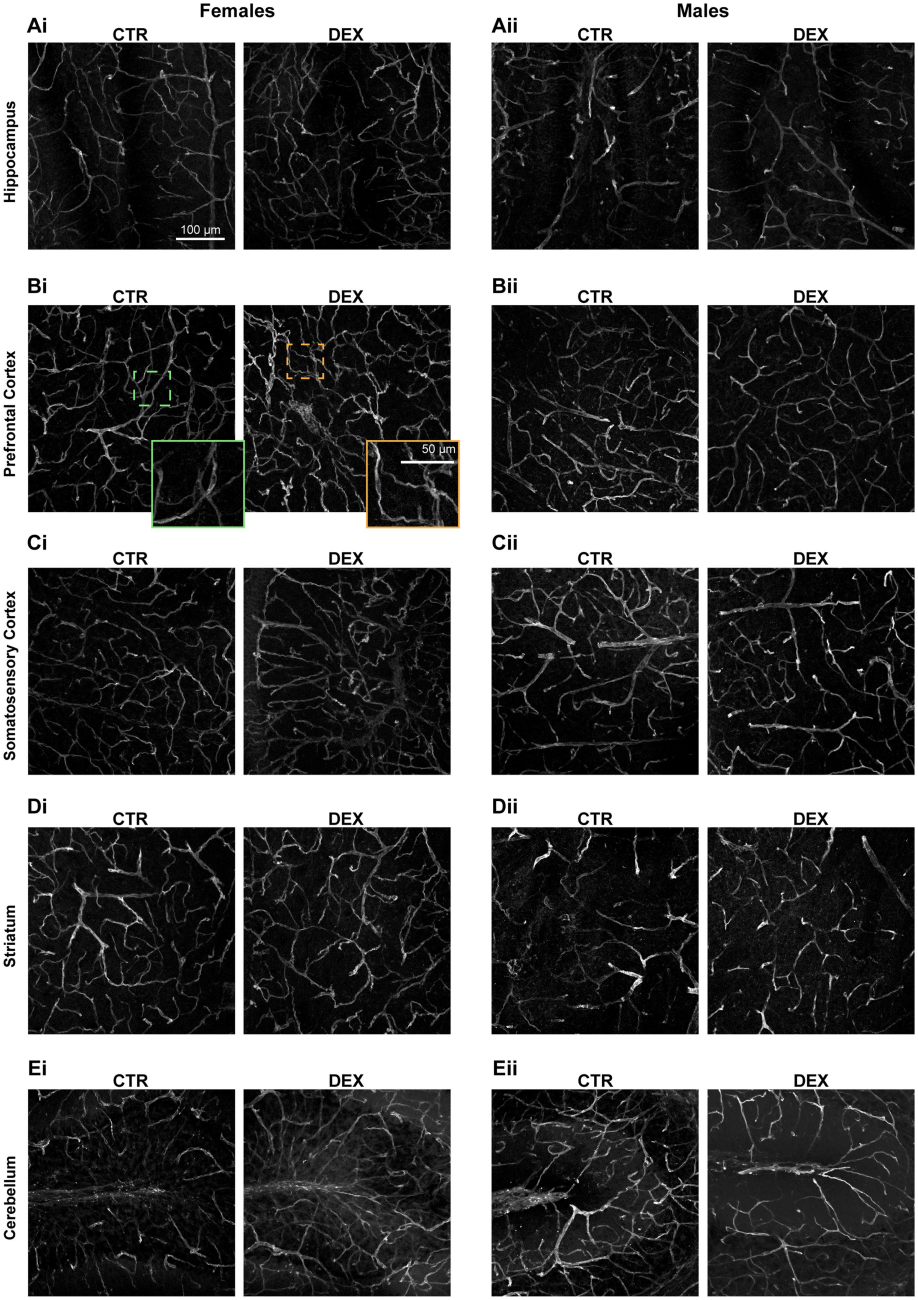
Confocal images of brain vasculature. Representative confocal images of vasculature in the hippocampus **(A)**, prefrontal cortex **(B)**, somatosensory cortex **(C)**, striatum **(D)**, and cerebellum **(E)** of female (i) and male (ii) offspring. Lectin staining is shown in green. An inset from Bi showing a magnified view of an example of a normal vessel (green) and a tortuous vessel (orange).

**Figure 5:**
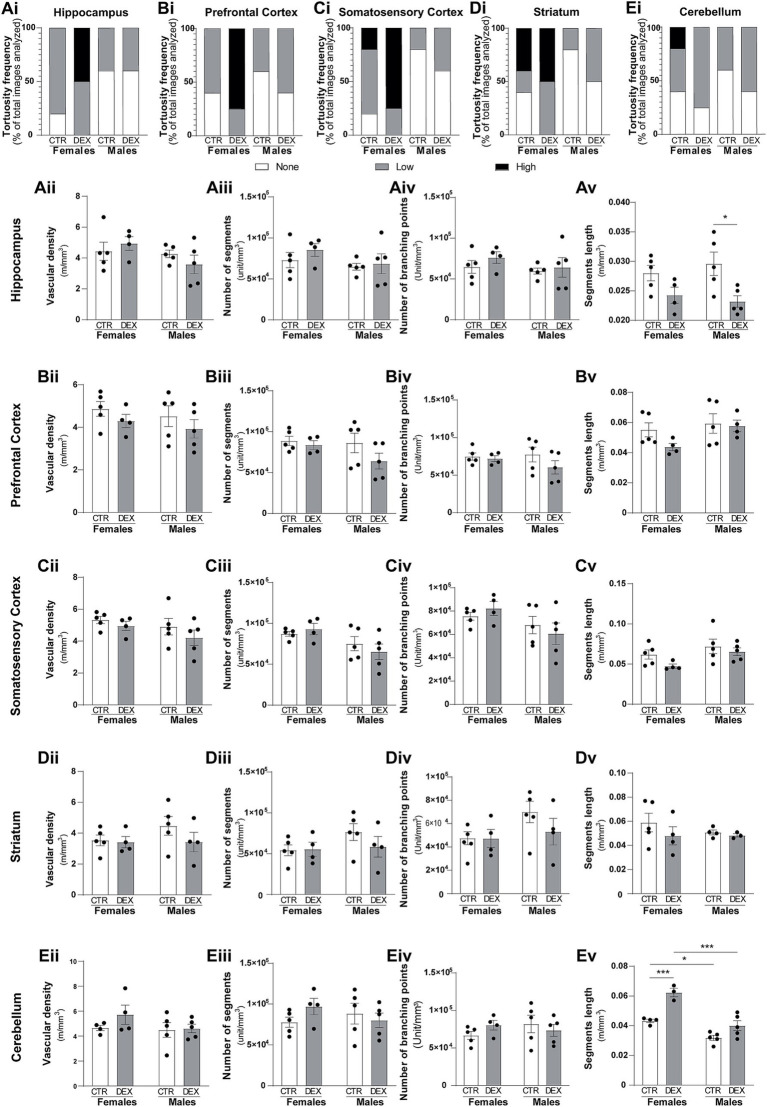
Sex-specific vascular effects of prenatal stress in infant offspring. Qualitative analysis of vessel tortuosity revealed an overall increase in DEX-treated female offspring **(Ai,Bi, Ci,Di,Ei)**. Quantitative analysis of microvessel morphology, including total vessel length, number of branches, branching points, and mean branch length, was performed for each brain region: hippocampus **(A)**, prefrontal cortex **(B)**, somatosensory cortex **(C)**, striatum **(D)**, and cerebellum **(E)**. Data are presented as mean ± SEM (*n* = 3–5 animals/group). *p < 0.05, ****p < 0.0001. Statistical significance was determined using a two-way ANOVA to assess the effects of prenatal stress (CTR vs. DEX) and sex (female vs. male).

A lower incidence of tortuous vessels was observed in males compared to female offspring. In general, male vessels generally exhibited none (50–80%) to low (20–50%) levels of tortuosity in physiological conditions across all brain regions. In female exposed to prenatal stress our analysis revealed an increase in highly tortuous vessels in most of the analyzed brain regions (Figures 4, 5Ai,Bi,Ci,Di). The hippocampus of females prenatally exposed to stress displayed a 50% increase in vessels with high tortuosity. Similarly, the prefrontal and somatosensory cortex exhibited increases of 75%, regarding highly tortuous vessels. Though the striatum also showed an increase, it was more modest, with only a 10% rise in highly tortuous vessels compared to CTR. Interestingly, the cerebellum was the only region where we did not observe noteworthy changes in tortuosity (Figures 4E, 5Ei). In male offspring prenatally exposed to DEX vessel tortuosity was less pronounced in all analyzed brain regions. The vascular density (total number of microvessels per mm^3^) seems to be similar in females and males across all the regions observed at P14 and remains largely unaffected by prenatal stress (Figures 5A–Eii). However, when we looked at the microvessel’s structure in detail, some alterations were noted. The number of segments and branching points within the hippocampus (Figures 5Aiii,iv), prefrontal cortex (Figures 5Biii,iv), striatum (Figures 5Diii,iv), and cerebellum (Figures 5Eiii,iv) remained largely unaffected in both sexes. No significant alterations were detected in segment length within the prefrontal cortex, somatosensory cortex, and striatum in both males and females prenatally exposed to stress (Figures 5Bv,Cv,Dv). No sex differences were observed in hippocampal segment length (Figure 5Av). However, antenatal DEX exposure led to a significant decrease in segment length in male offspring (*p* = 0.0132; Figure 5Av), while only a tendency was noted in females. At the cerebellum level, a sex dimorphism was registered under physiological conditions, with males exhibiting shorter vascular segments than females (*p* = 0.0113; Figure 5Ev). The prenatal DEX exposure significantly increased segment length in females (*p* = 0.0006; Figure 5Ev). While offspring males did not exhibit a significant response to treatment, a notable sex-dependent effect of treatment was observed (*p* = 0.0001; Figure 5Ev).

Overall, these results show a differential vulnerability of male and female brains to prenatal stress, with females exhibiting more pronounced alterations in vascular structure.

## 4 Discussion

Prenatal stress, a significant early life adversity, has the potential to imprint lasting effects on the central nervous system, increasing the risk of neuropsychiatric disorders later in life. It may disrupt the vasculogenesis, formation of the primary vascular plexus (Millage et al., 2017) and the astrocytic proliferation processes (Vivi and Di Benedetto, 2024) that take place during gestation. To the best of our knowledge, our findings reveal for the first time that prenatal DEX exposure mediates sex-specific changes in the gliovascular interface during infancy, with a dose that has been linked to neuropsychiatric symptoms in adulthood—specifically, schizophrenia-related behaviors in males and anxiety- and depressive-like behaviors in females—mirroring symptoms observed in humans (Rim et al., 2022).

The limited research conducted so far indicates that prenatal exposure to DEX may influence brain vasculature, hinting at region-specific alterations within the brain. Because of that, we analyzed several brain regions: hippocampus, prefrontal cortex, somatosensory cortex, striatum, and cerebellum. In female offspring, DEX increased vessel tortuosity displaying a corkscrew-like apparency in most regions, except the cerebellum, where segment length increased, while overall vascular structure remained largely unchanged. In male offspring, no major vascular changes were observed, aside from shortened segments in the hippocampus. This aligns with prior reports (Neigh et al., 2010) on DEX-induced decrease in hippocampal vasculature in adulthood males. The increase in tortuosity may be linked to alterations in the structure of endothelial cells lining the blood vessel walls, which have been reported in other studies investigating GC-induced endothelial cell dysfunction (Han, 2012), due to changes in tight junction proteins, which are crucial for maintaining the integrity of the BBB (Neuhaus et al., 2015). Likewise, existing studies explore how DEX affects endothelial cell activity, demonstrating increases in their proliferation and migration (Polytarchou and Papadimitriou, 2005; Wallerath et al., 2004). The vascular alterations observed can be manifested as a weakened vessel structure, further impacting vessel function, which could disrupt blood flow and homeostasis within these brain regions. Although not evaluated in this study, prenatal exposure to GC seems to induce vasodilation and alter vascular tone (Eiby et al., 2014).

Previous studies have shown that early life stress impacts astrocytic structure and function, particularly astrogliosis markers such as GFAP, GLT, and GLAST, in ways that may have implications later in life (Majcher-Maślanka et al., 2019; Zeng et al., 2020; Orso et al., 2023; Martisova et al., 2013). While astrocyte plasticity in adults has been associated to prenatal development (Shende et al., 2015), little is known about its impact during infancy or whether it represents an early effect. AQ4 within astrocytic endfeet plays a critical role in managing water dynamics within the brain. Located in the perivascular space, AQ4 connects astrocytes to the extracellular space around blood vessels, regulating water efflux and influx (Nakada et al., 2017). This helps maintain fluid balance within the BBB and supports cerebrospinal fluid flow (Nakada et al., 2017; Haj-Yasein et al., 2012; Igarashi et al., 2014). AQ4 function in adult brains has been well explored, however its role during development remains unclear (Fallier-Becker et al., 2014; Wen et al., 1999). Some studies suggest AQ4 helps reduce brain water content after birth, as brains lacking AQ4 (AQ4-KO mouse) take longer to lose excess of water (Li et al., 2013). Other studies show AQ4 expression strengthens around blood vessels by P14, coinciding with BBB formation in mice (Lunde et al., 2015) and contributing to BBB maturation (Li et al., 2013). We found no significant alterations in AQ4 protein localization or expression levels in male offspring exposed to prenatal stress. In females, only the prefrontal cortex and hippocampus were affected by DEX exposure, with an interesting phenomenon observed in the prefrontal cortex. In the hippocampus, prenatal stress significantly increased AQ4 levels without altering coverage. In the prefrontal cortex, AQ4 levels remained unchanged, but its co-localization with blood vessels increased, indicating a possible shift in localization without changes in vascular density or segment length. Very recently, researchers examined the effects of DEX exposure on astrocytic AQ4 levels and the clearance of extracellular proteins by astrocytes *in vitro* (Dias et al., 2025). Similar to our findings, DEX exposure did not induce significant changes in AQ4 density in cortical primary astrocytes cultures, but it did reduce *α*-syntrophin density. α-Syntrophin helps anchor AQ4 to the perivascular endfeet of astrocytes by interacting with dystrophin-associated complexes (Amiry-Moghaddam et al., 2003). This localization is important for efficient water transport and the clearance of extracellular fluid, including removal of waste through the glymphatic system. Thus, a decrease in α-syntrophin density likely disrupts AQ4 localization and consequently its activity. In fact, authors found that the clearance of proteins in astrocytic cultures was decreased after 24 h exposure to DEX (100 nM), suggesting that stress-mimicking conditions affect AQ4 function (Dias et al., 2025). Our results demonstrate that prenatal stress disrupts AQP4 localization and levels in the hippocampus and prefrontal cortex in females descendants, potentially delaying or impairing proper gliovascular maturation in these regions. Furthermore, this disruption may disturb the brain’s water balance, increasing the risk of conditions such as cerebral edema (fluid accumulation in the brain).This study highlights sex- and region-specific different responses to prenatal stress. Studies indicate that female and male mice present distinct brain region maturation processes driven by hormonal regulation (McCarthy, 2008). Early in development, males typically have larger brain regions, while females show more growth during the peripubertal phase (Qiu et al., 2018; Tkáč et al., 2023). At P14, we identified a wider somatosensory cortex and cerebellum in males at P14, with a similar but non-significant trend in the striatum and hippocampus. We also show that females are more affected than males by prenatal stress, with distinct brain regions exhibiting varying vulnerabilities. While males also displayed brain alterations, these were milder and primarily restricted to the hippocampus. In contrast, females showed significant changes in the hippocampus, prefrontal cortex, and cerebellum, while the somatosensory cortex and striatum appeared less affected. The hippocampus and prefrontal cortex are essential for cognitive functions such as learning, memory, planning, decision-making, and working memory (Szczepanski and Knight, 2014; Rubin et al., 2014). Dysfunctions in these processes can lead to behavioral alterations and elevate the risk of developing sex-specific mental disorders later in life (Sigurdsson and Duvarci, 2015; Anderson et al., 2009). The prefrontal cortex undergoes an extensive period of maturation, allowing for significant plasticity influenced by sex hormones (Markham et al., 2013). Similarly, the hippocampus follows a sexually dimorphic developmental pattern, contributing to cognitive and emotional differences between sexes (Meyer et al., 2017). Notably, its high corticosteroid receptor expression (Viho et al., 2022), may explain why this region is affected in both males and females.

Prenatal stress has also been reported to have sex-specific effects in humans, with girls showing a higher risk than boys (Graham et al., 2019). One possible explanation was demonstrated by RNA sequencing revealing sex-specific effects of GC on the placentas of E11.5 male and female mice. Most transcripts were downregulated in the placentas of females prenatally exposed to DEX, while in males remained unchanged or were upregulated (Lee et al., 2017). Sex hormones also play a role in the maturation of astrocyte populations and their function, demonstrated by perinatal androgen exposure that influenced the differentiation and number of astrocytes in various brain regions (Santos-Galindo et al., 2011). In this work, we did not find any major physiological difference at P14 among sexes regarding AQ4 expression and vascular endfeet coverage. Nevertheless, existing data shows lower female astrocyte density compared to males in response to stress (Orso et al., 2023). This increased vulnerability in females (Wellman et al., 2018; Goodwill et al., 2019) might be associated with heightened astrocytic sensitivity to stress. Previous studies (Rim et al., 2022) using our prenatal stress model with DEX exposure identified microglia sex-specific changes within these regions, potentially contributing to the susceptibility to psychiatric disorders like chronic anxiety and depression (Caetano et al., 2017; Gaspar et al., 2021). Interestingly, it was found that postnatal immune activation leads to sex-specific gliovascular interface abnormalities in the hippocampus, further suggesting that sex hormones influence the interaction between astrocytes and blood vessels during development (Ardalan et al., 2022). Brain vessels are a target for sex hormones (Bolte and Cordelières, 2006; Majcher-Maślanka et al., 2019; Zeng et al., 2020; Orso et al., 2023; Martisova et al., 2013; Shende et al., 2015). However, the fact that sex hormones exert time- and dose-dependent effects on tight junction expression, angiogenesis, and vascular tone (Haj-Yasein et al., 2012; Li et al., 2013) is not very well comprehended. Our study, therefore, reinforces the complex relationship between sex hormones, astrocytic function, and neurovascular development.

Understanding the sex-specific impact of early-life adversities on infant development is crucial, as it can reveal unique alterations that may allow for targeted interventions. In this study, we break new ground by investigating gliovascular responses to prenatal stress in both female and male offspring, a previously unexplored area. We identified sex-specific AQ4 alterations in DEX-exposed offspring. Given the link between prenatal stress and sex-specific mental health vulnerabilities, AQ4 may represent a promising molecular target for early-life therapeutic interventions.

## Data Availability

The raw data supporting the conclusions of this article will be made available by the authors, without undue reservation.
